# On the Affection Commonly Termed “Corn” in the Equine Foot

**Published:** 1880-04

**Authors:** James Hamill

**Affiliations:** Consulting Surgeon and Lecturer on the Art op Shoeing, as Applied to the Normal and Pathological Foot, Columbia Veterinary College; New York, 416 East 14th Street


### ART. VIII.—ON THE AFFECTION COMMONLY TERMED "CORN" IN THE EQUINE FOOT.

BY JAMES HAMILL, D. V. S.,

CONSULTING SURGEON AND LECTURER ON THE ART OF SHOEING, AS APPLIED TO THE NORMAL AND PATHOLOGICAL FOOT, COLUMBIA VETERINARY COLLEGE.

AMONG the numerous diseases of the horse's foot, that commonly denominated "CORN" is perhaps the most troublesome, one of the most frequent, most persistent, least understood and worst treated affections that falls to the lot of the veterinary surgeon or his humble assistant, the shoeing smith. Should the student of the subject refer to veterinary literature from the earliest times to the present day, he will only find confusion worse confounded as to the etiology, pathology and treatment of Corn. The subject is generally passed over glibly in a short paragraph, glittering with generalities or paradoxes, to illustrate which I append one such extract from a deservedly well-known work; it reads as follows: "Corn is an extravasation of blood into the meshes of the horn fibre from a rupture of the blood vessels which secrete the horn of the part, caused by external concussion or bruise, generally from improper shoeing." Now, a definition, to be a definition, should cover the *whole* subject to be defined; yet this definition fails to cover the case of the worst kind of corn we find; it defines only the very simplest variety. Blinding the reader to the dangers of a virulent disease, such a definition leads to an incalculable amount of damage to the horse, and on the grounds of humanity and science no effort should be spared to correct it.

To illustrate my meaning, I shall recount a typical case. A gentleman owns a valuable horse which begins to go a little lame; the animal is sent to the shoeing smith to have the foot examined; the examination reveals pain and inflammatory heat in one or both heels. The recesses formed by the inflection of the wall are pared out, and the well-known red-colored horn substance appears. "The horse has corns" is the dictum; then the stereotyped routine procedure for relieving "corn" is gone through with; the horse is a little better for a time, but it is not long before it has to be gone over again and again, only with this difference, that the relief is less and less and shorter and shorter with each repetition of the so-called curative procedure. The veterinary surgeon is now called in, gets the history of the case, and of course he must vary the treatment *a little;* the smith is ordered to pare out the part thoroughly, and to see if there is no pus. Probably the entire sole is thinned down, and perhaps, to please the whims of some new-fledged V. S., D. V. S., or even an M. R. C. V. S., the foot is put in either hot or cold water, rather according to the fancy of the one treating the animal than the requirements of the case; this is then followed by poulticing. If suppuration does not occur, two or three days of this treatment will generally relieve the animal. The veterinarian then orders the shoe to be eased or raised off the heel, the shoe to be tacked on, or driven very loosely; the animal walks apparently sound, and the doctor is, of course, complimented for his skill and ability. *Complimented for what?* For actually injuring the foot; perhaps for having inflicted almost irreparable damage!

To every observant student of the foot it must be clear that this paring of the sole and digging down in the heel causes a permanent injury to the parts within, and that the springing of the shoe off the heel means so much friction, and friction in its turn is so much more wasting of the hoof or horn structure, and ultimately death to some other part, due to the unequal pressure which usually bears most heavily on the weakest and thinnest portion of the wall, viz., the delicate elastic quarter, the very part we should protect most, as here the fibro cartilage portions take their rise, and it is here that the natural process of transition is going on from the cartilage to the ospedis, which, if injured by any means, exhibits inflammatory reaction immediately, and, extending to the entire cartilage, provokes premature and excessive ossification, with all its accompanying evils. Then, through the same unequal pressure, which is also exerted on the plantar surface of the hoof, we get the shrunken, narrow, wasted, disproportioned, in short, generally deformed foot—a foot imperfectly fitted to support weight, and detrimental to the animal's powers of speed and endurance, not to say anything about beauty. If those interested in preventing cruelty to the horse, as Mr. Henry Bergh, for example, and the society over which he presides, only knew half the miseries the horse suffers through this pernicious system of treatment, I fancy that he would try to get a bill through the Legislature for the extermination of Horse Doctors.

Lovers of the horse are forever harping on the suffering of the "noble horse," through man's inhumanity, but it is only the most trivial sufferings which they grapple with. But now comes the question: If this treatment is improper and injurious, what else can be done with a case of corn? I will say, better let it alone altogether, than treat it in this barbarous and unphilosophical manner. At any rate let us try first to learn what this pain in the heel, and the bloody infiltrated appearance of the horn is due to.

I admit that bad shoeing produces corns, and that any kind of shoeing will produce them; but such corns are generally of the mildest type, and can be easily eradicated. I deny, however, most decidedly, that shoeing is *always* a cause, and I claim that the assertions of those who say that unshod horses have no corns is false, or only applies to those on pasture or working but very little, and that on soft roads I have seen horses that were never shod * suffer from foot ailments, and among them corns occupied a prominent place. I am no radical advocate for any particular system of shoeing, and I am most ready to admit that no horse ever was, nor ever will be, properly shod with iron, and that no system of shoeing will give entire satisfaction in regard to the health of the foot, as long as circumstances compel us to use iron and steel as a protection to the hoof, as some of the natural functions of the hoof must be checked and others limited by such an unyielding appliance. But, does it follow, that because shod, all shod horses will have corns? Certainly not! Neither will all horses be free from corns! Then what is "corn"? It is not a "bruise"; it is not a rupture of blood vessels; neither is it horn rot, as some have described it. Nor is it caused by the impinging of the ospedis on the sensitive tissues lying between it and the horny sole, for in that case it would have to be looked for in different localities, at different ages of the horse. In the young foot the ospedis does not extend by its retrossal processes as far back as the situation of the "corn."

If it were a simple bruise, such as sometimes is produced through shoeing, the trouble would pass off gradually, as does also the change in color of the horn.

If it were a rupture of blood vessels, the shock to which it would be attributed must be something immense, and the extravation should occur very suddenly, for there is nothing in the anatomy of the parts and their general make-up, that predisposes to vascular rupture, even in the most energetic movements of the animal.

To go into the minute anatomy of the hoof would require a pretty large volume in itself; therefore, I can only say categorically, that rupture of the blood vessels supplying the horn cannot occur through any ordinary movement of the foot or limb, unless the union of the hoof with its foundation the ospedis,

---

* In the year 1875, during the performance at the great Roman hippodrome of P. T. Barnum, Esq., several animals that were used in the horse excercises of the ring, without shoes, suffered from laminitis of every grade.

through the elements of the keratogenic membrane, permitted of a rotatory movement.

There is *no* such rotation of the hoof on the organs within, and that disposes of the rupture theory.

The *union* of structures through the keratogenic membrane—though elastic—is yet so strong and complete, that rupture of blood vessels is impossible, without a lesion of such extent that it would make itself felt immediately. Of course this has reference to the foot in health.

It is to the diseased condition of this laminal structure that we must look for the distant cause of the worst cases of "corn," as well as of another condition akin to corn, known to horsemen *as* "*broken bar.*" *If these structures were not diseased, how could the descent of the processes of the ospedis, or of the cartilages occur, which by pressure on the structure between them and the horny sole, are the factors immediately concerned in producing both affections?*

The sole lying in the re-entering angle, between the bar and wall, could no more become affected by internal pressure than the anterior or middle portions, as long as the natural suspensory power was intact. This easily ascertainable fact appears to have been overlooked by nearly all English authorities on the foot.

In reality the pathological causation of corn differs from that producing laminitis, only in the locality at, and the extent to which the foot becomes affected.

As no one would claim that the shoe is always the cause of laminitis, as little could the shoe be blamed as the universal cause of corn. It will be observed that if we follow it from the start to its very worst termination, "corn" presents many analogies to laminitis. In the latter we have first the irritation, then the inflammation, which, if continued, destroys the adhesion of the horn to the vascular membrane. The suspensory power once lost, it can never be regained at that part, without a regeneration from above downward. By loss of the suspensory power, the ospedis descends on the villous tunie, or sensitive tissue secreting the sole; this pressure being prejudicial, it causes a local death of the tissue, horn secretion stops, and the natural exfoliation of the sole going on from its external surface, we get a time when a rupture of the part results. A distant result is that there being now no support for the ospedis at its anterior aspect, the entire pressure comes on the coronary cushion, the undue pressure of the chamber at the superior border of the wall causes *also* a diminution of the horn-secreting power of this part, and then occurs sloughing of the hoof, or the " breaking down " of laminitis.

In the first stage of inflammation of these different parts, there must be over activity of the horn-secreting membrane; this gives a greater cell proliferation, and the villi, under such circmstances, being more vascular, red blood corpuscles are plentifully transuded among the horn cells, and therefore in each portion of horn affected during the different stages of laminitis, we have the coloring matter of the blood more abundant, and with the very rapid formation going on, the horn is red in appearance, that color being deeper near the source of nutrition. Therefore, from the presence of a red color, it does not follow that we have had a rupture of blood vessels. If such had been the case the horn-secreting power of the membrane, instead of being increased, would be more likely to be destroyed. Death of this power is well marked, however, in the later stages of this disease.

All that is here stated in regard to laminitis occurs first at the most vascular part of the podophyllous tissue, namely, at the toe (where lie the hardest and strongest internal and external structures); it may then spread to the surrounding parts by contiguity, *et cetera.*

The part affected in " corn," is also the most vascular in the region of the *heels*, which is likewise pressed by the strong unyielding horn of the heel where it bends abruptly on itself, intensifying the pressure when inflammation of the internal parts exists with the same consequences, if the pressure be continued, that we get in the breaking down of laminitis, though in a less degree, owing to the difference in position of the laminae themselves, and a difference of the structures within. The causes which bring about inflammation of these vascular laminae of the heels are various. I know shoeing may be one, by indirectly acting on the part through a resulting contraction of the heels.

Then long rest, as well as the contrary, inordinate action from overwork, finally inflammation of the cartilages, may be communicated to the vascular structure. These structures are well known to be the seat of insidious inflammation through local as well as constitutional influences.

There is another and a more important case, viz., the contraction of the butress of the heel in a longitudinal direction, that is from above, downwards and forwards, bringing the inferior border of the inflection more under the suberincumbent weight of the body, than is natural. It is this that produces the much to be dreaded continued pressure on the laminae here situated.

Cases so produced are termed, " incurable corn," sometimes they are called " soft corns," and at others, " hollow corns," " gravel in the heels," and so on. The continued pressure produced in the manner related, destroys the growth of horn derived from the pedophylous tissue of the heels, and in the first stages we find the linar alba soft, or weakened, and if pressed upon by sand or grit, at its union with the sole, it will give way easily. This latter fact has given rise to the designation " gravel in the heels," and may progress to what is called " suppurating corn."

There is often seen, on paring the diseased part down, open spaces with probably a small amount of serous fluid ; these open spaces are the spaces between the lamina left by the non-secretion of horn. Death of this part may now go on until there is a necrotic space large enough to admit of turning the point of the search knife, without touching any substance ; *and yet no pus has at any time existed in the cavity.* At other times there is chronic inflammation, with probably necrosis of cartilage, and resulting deformity. In the majority of cases, this comes from the extreme paring resorted to by those in charge, and which I previously referred to, and which cannot be sufficiently condemned.

No sane man will pare a hoof to excess, nor dig through the sole to eradicate corn, unless there be pus in the part.

Paring thin has more than one undesirable sequel. All pared horn contracts on the surface pared ; this causes it to arch in the opposite direction ; the contraction is caused by loss of moisture, and can be readily demonstrated, whether the horn be dead horn of the foot, or live horn on it; the latter by every day experience. Then the paring of the entire sole would cause contraction of its external surface; this would draw the inferior border of the wall inward, and at the same time arch the sole in its center, thereby pushing its internal or upper surface against the living tissues within, and if not at once giving deformity, will be sure to produce evils of another and worse sort—that is, death of the secreting structure, or pressure on the navicular articulation.

"Broken bar" is the common term for the unhealthy secretion of horn in the middle portion of the bar; this is more generally due to overgrowth than to over paring but it is when the heels contract abruptly at their most posterior portion, that we have caused a greater deflection of the bar.

The deflection acts in such a manner as to send the middle portion of the bar upward and outward, causing undue pressure on the living structures, between it and the retrossal process of the ospedis, with irritative inflammation, death of the horn secreting power of the part corresponding to the same phenomenon noticed in laminitis and "corn," but more narrowly localized, causing it, when opening at the external surface, to look like the puncture from a nail, and such an opening, in reality due to a deep seated disorder, is disposed of by some sapient diagnostician with the phrase, "picked up a nail or wire."

In a proper study of the changes which occur in these diseases, their effect on the horn covering and mechanism of the foot may be found a key to their rational treatment, which subject I shall take up in the next number of the ARCHIVES.

NEW YORK, 416 EAST 14TH STREET.

